# Maternal age at first cesarean delivery related to adverse pregnancy outcomes in a second cesarean delivery: a multicenter, historical, cross-sectional cohort study

**DOI:** 10.1186/s12884-021-03608-9

**Published:** 2021-02-12

**Authors:** Shilei Bi, Lizi Zhang, Jingsi Chen, Minshan Huang, Lijun Huang, Shanshan Zeng, Yulian Li, Yingyu Liang, Jinping Jia, Suiwen Wen, Yinli Cao, Shaoshuai Wang, Xiaoyan Xu, Ling Feng, Xianlan Zhao, Yangyu Zhao, Qiying Zhu, Hongbo Qi, Lanzhen Zhang, Hongtian Li, Zhijian Wang, Lili Du, Dunjin Chen

**Affiliations:** 1 Department of Obstetrics and Gynecology, The Third Affiliated Hospital of Guangzhou Medical University, 63 Duobao Road, Guangzhou, 510150 Guangdong China; 2 Department of Obstetrics and Gynecology, Nanfang Hospital, Southern Medical University, 1838 Guangzhou Ave North, Guangzhou, 510515 Guangdong China; 3 Key Laboratory for Major Obstetric Diseases of Guangdong Province, Guangzhou, People’s Republic of China; 4 Key Laboratory of Reproduction and Genetics of Guangdong Higher Education Institutes, Guangzhou, People’s Republic of China; 5 Department of Obstetrics and Gynecology, Guangzhou Huadu District Maternal and Child Health Hospital, Guangzhou, China; 6 Department of Obstetrics and Gynecology, The Sixth Affiliated Hospital of Guangzhou Medical University, Qingyuan People’s Hospital, Guangzhou, China; 7 Department of Obstetrics and Gynecology, Northwest Women’s and Children’s Hospital, Xian, China; 8 Department of Obstetrics and Gynecology, Tongji Hospital, Tongji Medical College, Huazhong University of Science and Technology, Wuhan, China; 9 Department of Obstetrics and Gynecology, The First Affiliated Hospital of Zhengzhou University, Zhengzhou, China; 10 Department of Obstetrics and Gynecology, Peking University Third Hospital, Beijing, China; 11 Department of Obstetrics and Gynecology, The First Affiliated Hospital of Xinjiang Medical University, Urumqi, China; 12 Department of Obstetrics and Gynecology, The First Affiliated Hospital of Chongqing Medical University, Chongqing, China; 13 Department of Obstetrics and Gynecology, The Second Affiliated Hospital of Guangzhou Medical University, Guangzhou, China; 14 Institute of Reproductive and Child Health, National Health Commission Key Laboratory of Reproductive Health, Peking University Health Science Center, Beijing, China

**Keywords:** Maternal age, Pregnancy, Cesarean delivery, Complication, Adverse outcomes

## Abstract

**Background:**

To determine the effects of maternal age at first cesarean on maternal complications and adverse outcomes of pregnancy with the second cesarean.

**Methods:**

This was a multicenter, historical, cross-sectional cohort study involving singleton pregnancies ≥28 gestational weeks, with a history of 1 cesarean delivery, and who underwent a second cesarean between January and December 2017 at 11 public tertiary hospitals in 7 provinces of China. We analyzed the effects of maternal age at first cesarean on adverse outcomes of pregnancy in the second cesarean using multivariate logistic regression analysis.

**Results:**

The study consisted of 10,206 singleton pregnancies. Women were at first cesarean between 18 and 24, 25–29, 30–34, and ≥ 35 years of age; and numbered 2711, 5524, 1751, and 220 cases, respectively. Maternal age between 18 and 24 years at first cesarean increased the risk of placenta accreta spectrum (aOR, 1.499; 95% CI, 1.12–2.01), placenta previa (aOR, 1.349; 95% CI, 1.07–1.70), intrahepatic cholestasis of pregnancy (aOR, 1.947; 95% CI, 1.24–3.07), postpartum hemorrhage (aOR, 1.505; 95% CI, 1.05–2.16), and blood transfusion (aOR, 1.517; 95% CI, 1.21–1.91) in the second cesarean compared with the reference group (aged 25–29 years). In addition, maternal age ≥ 35 years at first cesarean was a risk factor for premature rupture of membranes (aOR, 1.556; 95% CI, 1.08–2.24), placental abruption (aOR, 6.464, 95% CI, 1.33–31.51), uterine rupture (aOR, 7.952; 95% CI, 1.43–44.10), puerperal infection (aOR, 6.864; 95% CI, 1.95–24.22), neonatal mild asphyxia (aOR, 4.339; 95% CI, 1.53–12.32), severe asphyxia (aOR, 18.439; 95% CI, 1.54–220.95), and admission to a neonatal intensive care unit (aOR, 2.825; 95% CI, 1.54–5.17) compared with the reference group (aged 25–29 years).

**Conclusions:**

Maternal age between 18 and 24 years or advanced maternal age at first cesarean was an independent risk factor for adverse maternal outcomes with the second cesarean. Advanced maternal age at the first cesarean specifically increased adverse neonatal outcomes with the second. Therefore, decisions as to whether to perform a first cesarean at a young or advanced maternal age must be critically evaluated.

**Supplementary Information:**

The online version contains supplementary material available at 10.1186/s12884-021-03608-9.

## Background

Age plays a significant role in infertility, pregnancy-related complications, and adverse obstetric and perinatal outcomes [1]. As the average human life span increases [2], the role of age in the development and outcome of diseases [2, 3] has also changed concomitantly. An apparent trend in obstetrics worldwide is that childbearing is being postponed to later age [4, 5]. Although advanced maternal age (AMA) is currently defined as maternal age 35 years or older at the time of delivery [6], some investigators define AMA as over 40 years [7, 8]. This trend is partially attributed to women’s pursuit of higher education, desire to have successful careers, and wish to attain financial stability [7, 9]. In addition, women delay childbearing by modifications to lifestyle (delayed marriage and increased rates of divorce) or due to underlying subfertility [10]. Effective contraception, developments in assisted reproductive technology, and multiparous women have also driven a shift toward postponing motherhood or bearing an additional child at a more advanced age [11]. The risks to women and newborns associated with AMA have therefore recently undergone greater scrutiny.

The rate of cesarean delivery (CD) has risen rapidly worldwide in recent years. In China, the cesarean rate increased from 28.8% in 2008 to 34.9% in 2014 [12], considerably above the World Health Organization (WHO)-recommended rate of 10–15% of total births [13]. It also appears that AMA is occurring concomitantly with the increasing rate in cesarean delivery. A systematic review with meta-analysis has demonstrated that AMA increased the risk of obstetric interventions such as CD [14]. Due to the previous “one-child” family planning policy in China, many women requested elective CD without valid medical indications for fear of a long and painful labor, or unplanned cesarean delivery after failure of vaginal birth or pelvic floor trauma [15]. In 2016, the enactment of the 2-child policy was expected to increase the rate of CDs as related to previous cesarean history [16].

Many investigators have reported on the relationship between maternal age and obstetric complications and/or adverse outcomes in pregnancy [17, 18]. Additionally, some studies have indicated an association between short inter-pregnancy interval and poor birth outcomes in the succeeding pregnancy—including preterm birth and extremely low birth weight—in women with advanced age [19, 20]. Qin et al. reported that AMA and previous cesarean section were both risk factors for adverse outcomes of pregnancy during second pregnancies [21]. However, little is known regarding the effects of age at first delivery on the subsequent pregnancy, especially the age at the first CD. To garner more insight into possible links, we herein analyzed the association between maternal age at first CD and complications and adverse outcomes of pregnancy in the second CD using data from 11 public tertiary hospitals within 7 provinces of China.

## Methods

### Study design

**Fig. 1:**
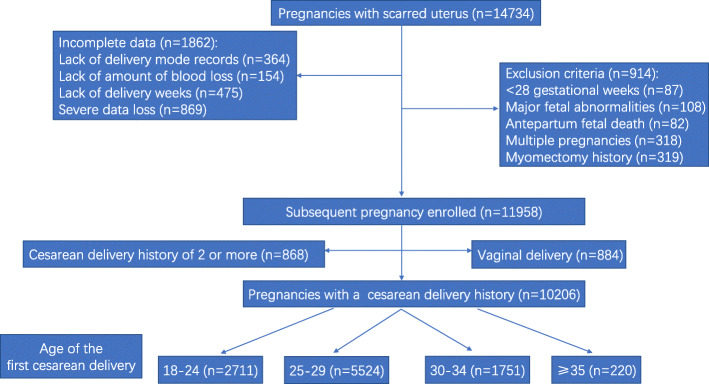
Flow diagram of the women’s enrollment process

This was a multicenter, historical, cross-sectional cohort study conducted at 11 public tertiary hospitals covering 7 provinces, municipalities, and autonomous regions within China (Guangdong, Beijing, Xinjiang, Shanxi, Henan, Hubei, and Chongqing). The cohort comprised 14,734 women with uterine scars who delivered again between January 2017 and December 2017. We selected women with singleton pregnancies at ≥28 gestational weeks, and with a CD history who underwent a repeat CD. Antepartum fetal death, major fetal congenital anomalies, a scarred uterus caused by myomectomy, and a history of 2 or more CDs were excluded. Women lacking their essential records—such as delivery mode or severe data loss—were also excluded. Figure 1 shows a flow diagram of the women’s enrollment process.

### Research methods

Data were obtained by chart review based on electronic medical records. Maternal clinical characteristics included maternal ages at the first and second CDs, gestational weeks, gravidity, parity, nationality, mode of conception (natural vs. assisted), source of pregnant women (“referral” meant that pregnant women were referred from low-level hospitals to tertiary hospitals. “hospital” meant that pregnant women delivered in a tertiary hospital from the beginning), body mass index (BMI) before pregnancy, number of months (interval) between the 2 CDs, sex of the offspring, and indications for the 2 CDs.

We reviewed the electronic medical records with regard to complications, and the following details were recorded: premature rupture of membranes (PROM), placenta previa (PP), placenta accreta spectrum disorders (PAS), placental abruption, idiopathic thrombocytopenic purpura (ITP), abnormal amniotic fluid (oligohydramnios and polyhydramnios), hypertension disorders, diabetes mellitus (DM), thyroid diseases (hypothyroidism and hyperthyroidism), and intrahepatic cholestasis of pregnancy (ICP).

We collected adverse maternal outcomes, including any of the following: postpartum hemorrhage (PPH), severe PPH, uterine rupture, disseminated intravascular coagulation (DIC), puerperal infection, hysterectomy, bladder injury, and blood transfusion. We defined PPH as a loss of ≥1000 ml of blood after cesarean delivery and severe PPH as the loss of ≥1500 ml of blood after cesarean delivery.

Adverse neonatal outcomes included prematurity (< 37 weeks), fetal growth restriction (FGR), mild asphyxia (1-min Apgar score < 8), severe asphyxia (1-min Apgar score < 4), neonatal complication, and admission to a neonatal intensive care unit (NICU). FGR is an estimated fetal weight that is less than the 10th percentile for gestational age. Neonatal complication included neonatal asphyxia, neonatal malformation and other complications (hemolysis, jaundice, congenital heart disease, meconium aspiration pneumonia, et al.).

Information regarding maternal and neonatal diseases was classified according to the WHO’s Classification of diseases (ICD)-10.

### Statistical analyses

We performed statistical analyses using SPSS v24.0 for Windows and R software (version 3.6.1). Missing values were imputed using a random forest algorithm. Missing data was listed in Supplementary Table S1. We examined whether quantitative data were normally distributed using the Kolmogorov-Smirnov test. Non-parametric continuous features are presented as medians and their corresponding interquartile ranges (IQR), and the Kruskal-Wallis test was applied for comparisons among multiple groups. Categorical variables are reported as frequencies (percentages), and the differences between groups were compared using the χ2 or Fisher exact-probability test in the case of small numbers, where appropriate. The associations between maternal age at first CD and each outcome were investigated by logistic regression analysis in 2 models: model 1 was adjusted for possible confounders—including gravidity, parity, BMI, assisted reproductive technology, and interval months; and with model 2 we explored possible explanations of the findings by adding mediating factors—i.e., maternal age at the second CD [19]. There is no official international definition of “advanced maternal age,” nor is there an “age interval” or reference group of maternal age [20]. According to most studies and guidelines, adolescent mothers have been excluded as a unique group. The rationale for our choice of a reference group (25–29 years) for this study was that the expected outcomes would be optimal for this age range, which also included the largest number of pregnancies. We also calculated crude odds ratios (ORs) and adjusted odds ratios (aORs), along with their 95% confidence intervals (CIs). Differences with *P*-values of < 0.05 were considered to be statistically significant.

## Results

**Fig. 2:**
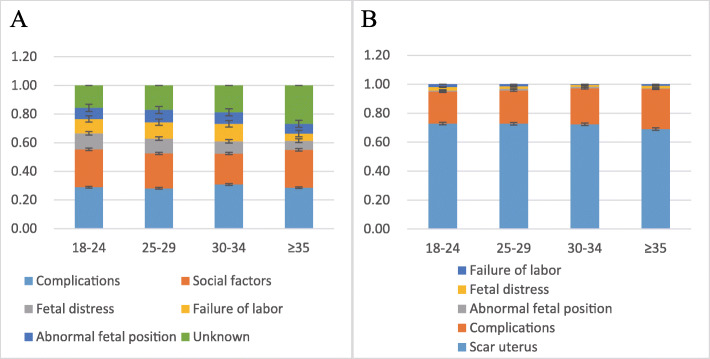
**a** Indications for the first CD in different age ranges. **b** Indications for the second CD in different age ranges

The present study consisted of 10,206 women with a history of CD who underwent a repeat CD. Women at their first CD were aged 18–24 years, 25–29 years, 30–34 years, and ≥ 35 years; and numbered 2711, 5524, 1751, and 220 cases, respectively. Figure 2 shows that the indications for the first CDs were principally pregnancy complications, fetal distress, failure of labor, abnormal fetal position, social factors, and unknown reasons. The indications for the second CDs were primarily uterine scarring, pregnancy complications, fetal distress, failure of labor, and abnormal fetal position. The compositional ratio of CD indications was similar for different age groups, and scarred uterus was the primary indication for a second CD.

Table 1 shows the general characteristics and potentially mediating factors for the women undergoing a consecutive CD—with the median values showing an increase, and the median interval between the 2 cesareans shortened in an almost continuously commensurate fashion relative to the age at first CD: 18–24 years of age (second CD at a median age of 29 years, with a median interval of 84 months); 25–29 years (33 years and 72 months); 30–34 years (36 years and 60 months); and ≥ 35 years (40 years and 48 months). In the group of women who were older than 35 years at their first CD, gravidity and parity increased; and more women underwent assisted reproductive technology prior to the second CD compared with other groups. A greater number of women between 18 and 24 years of age at their first CD tended to be either leaner (a BMI less than 18.5 kg/m^2^) or obese (a BMI above 30 kg/m^2^) with the second CD.

**Table 1:** General characteristics of women in various age ranges undergoing their second CD

Variables	18–24 (*n* = 2711)	25–29 (5524)	30–34 (*n* = 1751)	≥35 (220)	*P*
Gravidity					< 0.05
2	1212 (44.7%)	2804 (50.8%)	925 (52.8%)	79 (35.9%)	
3	829 (30.6%)	1606 (29.1%)	477 (27.2%)	58 (26.4%)	
≥ 4	670 (24.7%)	1114 (20.2%)	349 (19.9%)	83 (37.7%)	
Parity					< 0.05
1	2626 (96.9%)	5397 (97.7%)	1663 (95%)	178 (80.9%)	
2	73 (2.7%)	115 (2.1%)	77 (4.4%)	36 (16.4%)	
≥ 3	12 (0.4%)	12 (0.2%)	11 (0.6%)	6 (2.7%)	
Age at second CD	29 (27,33)	33 (31,36)	36 (35,38)	40 (38,42)	< 0.05
BMI (kg/m^2^)					< 0.05
< 18.5	266 (9.8%)	381 (6.9%)	88 (5%)	7 (3.2%)	
18.5–24.9	1812 (66.8%)	3783 (68.5%)	1208 (69%)	153 (69.5%)	
25–29.9	538 (19.8%)	1200 (21.7%)	394 (22.5%)	54 (24.5%)	
≥30	95 (3.5%)	160 (2.9%)	61 (3.5%)	6 (2.7%)	
Nationality					< 0.05
Han	2673 (98.6%)	5414 (98%)	1710 (97.7%)	208 (94.5%)	
Others	38 (1.4%)	110 (2%)	41 (2.3%)	12 (5.5%)	
Interval months	84 (53,120)	72 (48,108)	60 (40,84)	48 (32.25,60.75)	< 0.05
ART	72 (2.7%)	100 (1.8%)	71 (4.1%)	14 (6.4%)	< 0.05
Source					< 0.05
Hospital	2126 (78.4%)	4626 (83.7%)	1492 (85.2%)	185 (84.1%)	
Referral	585 (21.6%)	898 (16.3%)	259 (14.8%)	35 (15.9%)	
Gestational weeks	39 (37,39)	39 (38,39)	39 (38,39)	39 (37.25,39)	< 0.05
Sex					0.951
Male	1477 (54.5%)	3047 (55.2%)	960 (54.8%)	121 (55%)	
Female	1234 (45.5%)	2477 (44.8%)	791 (45.2%)	99 (45%)	

*BMI* Body mass index, *ART* Assisted reproductive technology, *CD* Cesarean delivery

The effects of maternal age at first CD on maternal complications with the second CD were the principal findings of our study (model 2). A maternal age of 18–24 years at the first CD increased the risk of PAS (aOR, 1.499; 95% CI, 1.12–2.01), PP (aOR, 1.349; 95% CI, 1.07–1.70), and ICP (aOR, 1.947; 95% CI, 1.24–3.07) at the second CD compared with the reference group (aged 25–29 years). By comparison, maternal age ≥ 35 years at first CD was a risk factor for PROM (aOR, 1.556; 95% CI, 1.08–2.24) and placental abruption (aOR, 6.464; 95% CI, 1.33–31.51) (Table 2).

**Table 2:** Maternal complications of a second CD for women of different age ranges at the first CD

Variables	Age	N (%)	OR (95%CI)	Model 1	Model 2
PAS	18–24	226 (8.3)	1.391 (1.17–1.66)	1.369 (1.15–1.63)	1.499 (1.12–2.01)
	25–29	339 (6.1)	1	1	1
	30–34	103 (5.9)	0.956 (0.76–1.20)	0.888 (0.70–1.12)	0.815 (0.59–1.12)
	≥35	25 (11.4)	1.961 (1.28–3.02)	1.387 (0.88–2.18)	1.149 (0.59–2.24)
PP	18–25	390 (14.4)	1.322 (1.15–1.51)	1.335 (1.16–1.53)	1.349 (1.07–1.70)
	25–29	623 (11.3)	1	1	1
	30–34	193 (11)	0.975 (0.82–1.16)	0.879 (0.74–1.05)	0.87 (0.68–1.11)
	≥35	36 (16.4)	1.539 (1.07–2.22)	1.063 (0.72–1.56)	1.04 (0.61–1.78)
Placental abruption	18–24	31 (1.1)	1.32 (0.84–2.08)	1.356 (0.86–2.14)	0.793 (0.37–1.69)
	25–29	48 (0.9)	1	1	1
	30–34	19 (1.1)	1.251 (0.73–2.14)	1.252 (0.73–2.16)	2.055 (0.95--4.44)
	≥35	4 (1.8)	2.113 (0.76–5.91)	2.202 (0.76–6.38)	6.464 (1.33–31.51)
Abnormal amniotic fluid	18–24	299 (11)	1.075 (0.93–1.25)	1.108 (0.96–1.29)	0.974 (0.76–1.25)
	25–29	571 (10.3)	1	1	1
	30–34	165 (9.4)	0.902 (0.75–1.08)	0.823 (0.68–0.99)	0.932 (0.72–1.21)
	≥35	20 (9.1)	0.867 (0.54–1.39)	0.73 (0.46–1.17)	0.971 (0.52–1.81)
Hypertension	18–24	296 (10.9)	1.08 (0.93–1.25)	1.093 (0.94–1.27)	1.044 (0.82–1.34)
	25–29	563 (10.2)	1	1	1
	30–34	190 (10.9)	1.073 (0.90–1.28)	1.022 (0.86–1.22)	1.076 (0.83–1.39)
	≥35	32 (14.5)	1.5 (1.02–2.20)	1.297 (0.87–1.93)	1.456 (0.83–2.57)
DM	18–24	579 (21.4)	0.853 (0.76–0.95)	0.827 (0.74–0.93)	1.091 (0.91–1.31)
	25–29	1334 (24.1)	1	1	1
	30–34	485 (27.7)	1.203 (1.07–1.36)	1.275 (1.13–1.44)	0.976 (0.81–1.17)
	≥35	72 (32.7)	1.528 (1.15–2.04)	1.63 (1.21–2.19)	0.907 (0.60–1.37)
PROM	18–24	333 (12.3)	0.915 (0.80–1.05)	0.918 (0.80–1.06)	0.905 (0.78–1.05)
	25–29	733 (13.3)	1	1	1
	30–34	238 (13.6)	1.028 (0.88–1.20)	1.013 (0.86–1.19)	1.022 (0.87–1.20)
	≥35	42 (19.1)	1.542 (1.09–2.18)	1.529 (1.07–2.18)	1.556 (1.08–2.24)
ICP	18–24	37 (1.4)	1.946 (1.24–3.06)	1.927 (1.23–3.03)	1.947 (1.24–3.07)
	25–29	39 (0.7)	1	1	1
	30–34	11 (0.6)	0.889 (0.45–1.74)	0.897 (0.46–1.76)	0.892 (0.45–1.76)
	≥35	2 (0.9)	1.29 (0.31–5.38)	1.317 (0.32–5.49)	1.321 (0.31–5.65)
ITP	18–24	55 (2)	1.465 (1.03–2.08)	1.565 (1.10–2.22)	1.057 (0.70–1.60)
	25–29	77 (1.4)	1	1	1
	30–34	15 (0.9)	0.611 (0.35–1.07)	0.528 (0.30–0.99)	0.779 (0.44–1.39)
	≥35	1 (0.5)	0.323 (0.05–2.33)	0.246 (0.03–1.79)	0.58 (0.08–4.34)
Thyroid disease	18–24	69 (2.5)	0.651 (0.49–0.86)	0.632 (0.48–0.83)	1.031 (0.68–1.57)
	25–29	213 (3.9)	1	1	1
	30–34	88 (5)	1.319 (1.02–1.70)	1.406 (1.08–1.82)	0.907 (0.61–1.35)
	≥35	12 (5.5)	1.439 (0.79–2.62)	1.605 (0.88–2.94)	0.633 (0.26–1.57)

*PAS* Placenta accreta spectrum disorders, *PP* Placenta previa, *DM* Diabetes mellitus, *PROM* Premature rupture of membranes, *ICP* Intrahepatic cholestasis of pregnancy, *ITP* Idiopathic thrombocytopenic purpura, *OR* Odds ratios, *CI* Confidence interval

Model 1: adjusted factors included gravidity, parity, BMI, ART, and interval months

Model 2: adjusted factors included gravidity, parity, BMI, ART, interval months, and maternal age at second CD

We further explored the effects of maternal age at first CD on adverse maternal and neonatal outcomes with the second CD (Tables 3 and 4). A maternal age of 18–24 years at the first CD increased the risk of PPH (aOR, 1.505; 95% CI, 1.05–2.16) and blood transfusion (aOR, 1.517; 95% CI, 1.21–1.91) with the second CD compared with the reference group (aged 25–29 years), while maternal age ≥ 35 years at first CD was a risk factor for uterine rupture (aOR, 7.952; 95% CI, 1.43–44.10) and puerperal infection (aOR, 6.864; 95% CI, 1.95–24.22).

**Table 3:** Adverse maternal outcomes for the second CD of women in different age ranges at first CD

Variables	Age	N (%)	OR (95%CI)	Model 1	Model 2
PPH	18–24	155 (5.7)	1.581 (1.28–1.96)	1.521 (1.23–1.89)	1.505 (1.05–2.16)
	25–29	204 (3.7)	1	1	1
	30–34	64 (3.7)	0.989 (0.74–1.32)	0.971 (0.73–1.29)	0.974 (0.65–1.46)
	≥35	9 (4.1)	1.112 (0.56–2.20)	0.913 (0.46–1.82)	0.88 (0.35–2.23)
Severe PPH	18–24	79 (2.9)	1.45 (1.08–1.94)	1.383 (1.03–1.86)	1.188 (0.72–1.95)
	25–29	112 (2)	1	1	1
	30–34	28 (1.6)	0.785 (0.52–1.19)	0.729 (0.48–1.11)	0.858 (0.49–1.52)
	≥35	4 (1.8)	0.895 (0.33–2.45)	0.552 (0.20–1.57)	0.793 (0.21–3.06)
Blood transfusion	18–24	182 (6.7)	1.76 (1.44–2.16)	1.745 (1.42–2.14)	1.517 (1.21–1.91)
	25–29	217 (3.9)	1	1	1
	30–34	60 (3.4)	0.868 (0.65–1.16)	0.816 (0.61–1.10)	0.926 (0.68–1.25)
	≥35	10 (4.5)	1.165 (0.61–2.23)	0.853 (0.44–1.67)	1.127 (0.57–2.23)
DIC	18–24	3 (0.1)	3.059 (0.51–18.32)	2.987 (0.49–18.22)	1.501 (0.08–26.37)
	25–29	2 (0)	1	1	1
	30–34	0	0	0.806 (0.61–0.10)	0
	≥35	1 (0.5)	12.607 (1.14–139.57)	13.95 (1.05–185.44)	51.8 (0.32–8409.68)
Uterine rupture	18–24	11 (0.4)	2.497 (1.03–6.03)	2.579 (1.06–6.25)	2.034 (0.75–5.49)
	25–29	9 (0.2)	1	1	1
	30–34	4 (0.2)	1.403 (0.43–4.56)	1.294 (0.39–4.27)	1.626 (0.48–5.53)
	≥35	2 (0.9)	5.622 (1.21–26.18)	5.002 (1.01–24.73)	7.952 (1.43–44.10)
Hysterectomy	18–24	2 (0.1)	0.679 (0.14–3.37)	0.304 (0.06–1.69)	0.387 (0.03–4.50)
	25–29	6 (0.1)	1	1	1
	30–34	1 (0.1)	0.526 (0.06–4.37)	1.208 (0.13–10.90)	0.939 (0.05–16.67)
	≥35	0	0	0.806 (0.61–0.10)	0
Bladder injury	18–24	43 (1.6)	1.111 (0.76–1.62)	1.03 (0.71–1.50)	1.199 (0.80–1.81)
	25–29	79 (1.4)	1	1	1
	30–34	31 (1.8)	1.242 (0.82–1.89)	1.3 (0.85–2.00)	1.128 (0.73–1.74)
	≥35	3 (1.4)	0.953 (0.30–3.04)	0.904 (0.28–2.94)	0.661 (0.20–2.19)
Puerperal infection	18–24	10 (0.4)	0.97 (0.46–2.06)	0.986 (0.46–2.10)	0.737 (0.32–1.72)
	25–29	21 (0.4)	1	1	1
	30–34	4 (0.2)	0.6 (0.21–1.75)	0.585 (0.20–1.71)	0.68 (0.23–2.06)
	≥35	4 (1.8)	4.853 (1.65–14.26)	4.634 (1.58–13.64)	6.864 (1.95–24.22)

*PPH* Postpartum hemorrhage, *DIC* Disseminated intravascular coagulation, *OR* Odds ratio, *CI* Confidence interval;

Model 1: adjusted factors included gravidity, parity, BMI, ART, and interval months

Model 2: adjusted factors included gravidity, parity, BMI, ART, interval months, and maternal age at second CD

**Table 4:** Neonatal outcomes of the second CD for woman of different age ranges at first CD

Variables	Age	N (%)	OR (95%CI)	Model 1	Model 2
FGR	18–24	72 (2.7)	2.037 (1.45–2.83)	2.061 (1.48–2.86)	1.223 (0.70–2.14)
	25–29	73 (1.3)	1	1	1
	30–34	24 (1.4)	1.038 (0.65–1.65)	1.025 (0.64–1.63)	1.641 (0.88–3.08)
	≥35	1 (0.5)	0.341 (0.05–2.46)	0.332 (0.05–2.40)	0.857 (0.10–7.61)
Mild asphyxia	18–24	102 (3.8)	1.585 (1.22–2.06)	1.599 (1.23–2.08)	0.882 (0.57–1.38)
	25–29	133 (2.4)	1	1	1
	30–34	40 (2.3)	0.948 (0.66–1.36)	0.891 (0.62–1.28)	1.577 (0.97–2.57)
	≥35	8 (3.6)	1.53 (0.74–3.16)	1.159 (0.55–2.46)	4.339 (1.53–12.32)
Severe asphyxia	18–24	10 (0.4)	1.019 (0.48–2.18)	1.014 (0.47–2.17)	0.317 (0.09–1.15)
	25–29	20 (0.4)	1	1	1
	30–34	2 (0.1)	0.315 (0.07–1.35)	0.292 (0.07–1.26)	0.831 (0.15–4.66)
	≥35	2 (0.9)	2.525 (0.59–10.87)	1.776 (0.38–8.26)	18.439 (1.54–220.95)
Preterm	18–24	393 (14.5)	1.442 (1.26–1.66)	1.457 (1.27–1.67)	1.092 (0.87–1.38)
	25–29	581 (10.5)	1	1	1
	30–34	172 (9.8)	0.927 (0.78–1.11)	0.872 (0.73–1.05)	1.143 (0.89–1.47)
	≥35	22 (10)	0.945 (0.60–1.48)	0.77 (0.49–1.22)	1.393 (0.77–2.53)
Neonatal complications	18–24	91 (3.4)	1.453 (1.11–1.91)	1.469 (1.12–1.93)	1.131 (0.72–1.79)
	25–29	129 (2.3)	1	1	1
	30–34	43 (2.5)	1.053 (0.74–1.49)	1.014 (0.71–1.44)	1.303 (0.80–2.13)
	≥35	8 (3.6)	1.578 (0.76–3.27)	1.356 (0.65–2.85)	2.343 (0.82–6.74)
NICU	18–24	284 (10.5)	1.426 (1.22–1.67)	1.425 (1.22–1.67)	1.036 (0.79–1.35)
	25–29	419 (7.6)	1	1	1
	30–34	118 (6.7)	0.88 (0.71–1.09)	0.841 (0.68–1.04)	1.127 (0.84–1.51)
	≥35	29 (13.2)	1.85 (1.24–2.77)	1.481 (0.98–2.25)	2.825 (1.54–5.17)

*FGR* Fetal growth restriction, *NICU* Neonatal intensive care unit, *OR* Odds ratio, *CI* Confidence interval;

Model 1: adjusted factors included gravidity, parity, BMI, ART, and interval months

Model 2: adjusted factors included gravidity, parity, BMI, ART, interval months, and maternal age at second CD

AMA at the first CD increased adverse neonatal outcomes with the second CD, including mild asphyxia (aOR, 4.339; 95% CI, 1.53–12.32), severe asphyxia (aOR, 18.439; 95% CI, 1.54–220.95), and admission to the NICU (aOR, 2.825; 95% CI, 1.54–5.17) compared with the reference group (aged 25–29 years).

After adjusting for maternal age at the second CD, maternal age ≥ 35 years at first CD did not influence the incidence of DIC (model 1: aOR, 13.951; 95% CI, 1.05–185.44; model 2: aOR, 51.8; 95% CI, 0.32–8409.68). In addition, the odds of ITP (model 1: aOR, 1.565; 95% CI, 1.10–2.22; model 2: aOR, 1.057; 95% CI, 0.70–1.60), severe PPH (model 1: aOR, 1.383; 95% CI, 1.03–1.86; model 2: aOR, 1.188; 95% CI, 0.72–1.95), uterine rupture (model 1: aOR, 2.579; 95% CI, 1.06–6.25; model 2: aOR, 2.034; 95% CI, 0.75–5.49), FGR (model 1: aOR, 2.061; 95% CI, 1.48–2.86; model 2: aOR, 1.223; 95% CI, 0.70–2.14), mild asphyxia (model 1: aOR, 0.87–1.38; model 2: aOR, 0.882, 95% CI, 0.57–1.38), neonatal complications (model 1: aOR, 1.469; 95% CI, 1.12–1.93; model 2: aOR, 1.131; 95% CI, 0.72–1.79), and admission to the NICU (model 1: aOR, 1.425; 95% CI, 1.22–1.67; model 2: aOR, 1.036; 95% CI, 0.79–1.35) for women between 18 and 24 years of age were not statistically significant with the second CD after adjusting for maternal age.

## Discussion

We found that maternal age at the first cesarean delivery was associated with obstetric complications and adverse outcomes of pregnancy with the second cesarean delivery. Compared with the reference group (25–29 years), maternal age between 18 and 24 years at the first CD increased the risk of PAS, PP, ICP, PPH, and blood transfusion with the second CD. In addition, maternal age ≥ 35 years at first CD was a risk factor for PROM, placental abruption, uterine rupture, puerperal infection, mild neonatal asphyxia, severe asphyxia, and admission to the NICU. We found that ages between 25 and 34 years constituted optimal times for the first CD from the perspective of outcomes with the second CD. And the study can be used for counseling AMA or young patients about possible adverse outcome of a second CD.

We noted in our study that, intriguingly, maternal age between 18 and 24 years at first CD increased the risk of PAS, PP, ICP, PPH, and blood transfusion with the second CD. In the United States, the highest rate of unintended pregnancy occurs among young adult women in their early twenties (18–24 years). Young adult women are likely to be unmarried, poorer, cohabiting, or participating in the labor force without having completed a college education; and it has been reported that unintended pregnancies are associated with adverse infant and maternal health outcomes [21, 22]. Additionally, in young women, unhealthy behaviors such as tobacco use and high body mass index were independent risks for PAS and PP [23]; and postpartum endometritis due to ignorance of postpartum care and poor postpartum recovery was associated with PAS disorders in subsequent pregnancies [23]. PAS and PP were the principal reasons for PPH and blood transfusion with the second CD [24]. A history of illicit drug use and viral hepatitis of any type increased the risk of ICP, with the recurrence rates for ICP ranging from 40 to 92% [25].

In our study, maternal age ≥ 35 years at first CD was a substantial risk factor for PROM, placental abruption, uterine rupture, and puerperal infection. A large proportion of AMA pregnancies were undesirable and unplanned—with short interpregnancy intervals, insufficient recovery of the uterus, physiologic stresses of a previous pregnancy [26], and reduced resistance—all potentially related to adverse pregnancy outcomes. Furthermore, it was alarming that maternal age ≥ 35 years at first CD was associated with a 4-fold increase in neonatal mild asphyxia, 18-fold increase in severe asphyxia, and 2-fold increase in NICU admission. Numerous investigators have reported that AMA was a risk factor for preterm delivery [27], low birthweight [28], fetal chromosomal abnormalities [29], and fetal death [30]. AMA was also associated with pre-eclampsia, gestational diabetes, PROM, venous thromboembolism, and the use of assisted conception [17, 26]—which would likely increase adverse neonatal outcomes.

In China, the rate of CD increased from 28.8% in 2008 to 34.9% in 2014 [12]. When we analyzed the indications for the 2 CDs, we found that the compositional ratios of CD indications were similar for the different age groups. The primary indications for the first CDs were pregnancy complications and social factors, while for the second CDs they were scarred uterus and pregnancy complications. In order to reduce the rate of CD, vaginal birth after 1 CD without contraindications has been recommended in China. However, the proportion of trials of vaginal birth after 1 CS was lower (9.1%) in our previous study compared with that in other countries (75.6% in Israel [31] and 70% in France) [32]. In our study, the rate of trials of vaginal birth among women between 18 and 24 years of age at their first CD was higher than for women who were ≥ 35 years of age (14.9% vs. 4.5%, respectively). Our best advice is to avoid unnecessary CDs, encourage women to have their children at an appropriate age, and propose that more women choose a trial of labor after a CD so as to decrease excess risks. This paper can be used for counseling AMA or young patients about possible adverse outcome of a second CD.

### Strengths and limitations

This study possesses several strengths. First, our study was based upon the multicenter database that encompassed 11 public tertiary hospitals; and it covered 7 provinces, municipalities, and autonomous regions within China in 2017, effectively avoiding the selection bias of single-center and small-sample studies. To some extent, these data can therefore be generalizable to more heterogeneous populations. Second, this was the first study of its kind on the effect of maternal age at first CD on the complications and adverse outcomes of the second CD. However, this study also has several limitations. This was a historical cohort study, and data from different centers were not always ideal and complete; some missing values were therefore imputed using the random forest algorithm. The number of women at their first CD with age ≥ 35 years was relatively small. Further repeated studies are needed.

## Conclusions

In conclusion, this multicenter, historical, cross-sectional cohort study of singleton pregnancies showed that maternal age between 18 and 24 years at first CD increased the risks for PAS, PP, ICP, PPH, and blood transfusion with the second CD. Maternal age ≥ 35 years at first CD was a risk factor for PROM, placental abruption, uterine rupture, puerperal infection, neonatal mild asphyxia, severe asphyxia, and admission to a NICU. The underlying mechanism(s) governing these relationships are unclear, and therefore further studies are needed to confirm that both young and advanced maternal ages at first CD are risk factors for many serious complications and adverse pregnancy outcomes at the subsequent CD.

## Supplementary Information

**Additional file 1: Table S1.** Missingness table

## Data Availability

The datasets used and/or analyzed during the current study are available from the corresponding author on reasonable request.

## Abbreviations

BMI: Body mass index
CD: Cesarean delivery
aOR: Adjusted odds ratios
CI: Confidence interval
PAS: Placenta accreta spectrum
PP: Placenta previa
ICP: Intrahepatic cholestasis of pregnancy
PPH: Postpartum hemorrhage
PROM: Premature rupture of membrane
NICU: Neonatal intensive care unit
AMA: Advanced maternal age
ITP: Idiopathic thrombocytopenic purpura
DM: Diabetes mellitus
DIC: Disseminated intravascular coagulation
FGR: Fetal growth restriction
