# Supplementary material for: Maternal age at first cesarean delivery related to adverse pregnancy outcomes in a second cesarean delivery: a multicenter, historical, cross-sectional cohort study
Source: BMC Pregnancy Childbirth. 2021 Feb 12;21:126. doi: 10.1186/s12884-021-03608-9 (PMC7881558; doi:10.1186/s12884-021-03608-9)
Supplement: Supplementary file 1 — Additional file 1: Table S1. Missingness table [file 12884_2021_3608_MOESM1_ESM.docx]

Table S1 Missingness table

| Variables | N (%) |
| --- | --- |
| Parity | 14 (0.13) |
| BMI | 820 (8) |
| Nationality | 21 (0.2) |
| Interval months | 49 (0.48) |
| ART | 57 (0.56) |
| Source | 52 (0.51) |
| PAS | 627 (6.1) |
| PP | 603 (5.9) |
| Placenta abruption | 655 (6.4) |
| Abnormal amniotic fluid | 649 (6.4) |
| Hypertension | 629 (6.2) |
| DM | 603 (5.9) |
| PROM | 514 (5) |
